# Perceptions of medical students regarding brain drain and its effects on Pakistan’s socio-medical conditions: A cross-sectional study

**DOI:** 10.12669/pjms.39.2.7139

**Published:** 2023

**Authors:** Fizzah Nadir, Hassan Sardar, Husnain Ahmad

**Affiliations:** 1 Dr. Fizzah Nadir, MBBS., Punjab Medical College, Faisalabad, Pakistan; 2 Dr. Hassan Sardar, MBBS., Punjab Medical College, Faisalabad, Pakistan; 3 Dr. Husnain Ahmad, MBBS., Punjab Medical College, Faisalabad, Pakistan

**Keywords:** Brain Drain, Immigration, Medical students, Pakistan socio-medical condition

## Abstract

**Background and Objective:**

The term “brain drain” refers to the exodus of educated or professional individuals to another nation, industry, or field, typically in search of a higher salary or living standard. Our objective was to look into the reasons why medical students leave after graduation, how it affects Pakistan’s socio-medical situation, and what may be done to address it.

**Methods:**

After receiving approval from the ethical committee, a cross-sectional survey was carried out among 420 undergraduate medical students of both genders at two different medical colleges in Pakistan for the current academic year 2021–2022. The core data was gathered via an organized questionnaire. The non-probability sampling method was employed to collect the sample. The data were analyzed with SPSS software.

**Result:**

Out of 420 medical students of both genders, 140 (33.3%) plan to leave Pakistan after graduation, while 280 (66.66%) want to pursue a career in Pakistan. Additionally, when asked about the amenities offered to Pakistani doctors throughout their training, the vast majority of medical students expressed satisfaction with living in Pakistan. However, among medical students who prefer to immigrate, the United States was at the top of their list. Although there are numerous reasons that affect doctors’ immigration choices, the majority of students think that poor pay and long working hours are the main ones that lead to poor patient management and inadequate training. This may be prevented by improving the pay and service structure.

**Conclusion:**

According to this survey, one in three medical students aspires to move overseas after graduation owing to a lack of resources and ineffective management, which has been adversely affecting Pakistan’s socio-medical situation. As a result, it is important for Pakistan’s health officials to launch campaigns to address the issues faced by medical students and physicians in order to prevent brain drain.

## INTRODUCTION

The term “brain drain” refers to the migration of educated or professional individuals to another nation, economic sector, or profession, typically in search of better salary or living conditions.^1^ The United States, the United Kingdom, Canada, and Australia are the four high-income countries (HICs) where around 56% of all doctors who migrate internationally (IMGs) are relocating from developing nations. Furthermore, just three nations India, Pakistan, and the Philippines are home to 45% of all IMGs.^2^ However, in terms of remittances sent home and cooperation in education and research, physician migration may have certain benefits that can be used to prove “brain gain”.^3^ According to the Bureau of Emigration and Overseas Employment, between 1000 and 1500 physicians leave the country each year, while 10 to 15 percent of them come back. This results in a net migration of 900 to 1,275 physicians. The most intellectually and socially gifted doctors are typically those who migrate, and they have the capacity to organize and transform Pakistan’s underdeveloped healthcare system.^4^,^5^

There are several significant “push factors” that contribute to the exodus of doctors from Pakistan such as low income, poor living standards, lack of training opportunities, and many more. On the other side, the pull factors, are the severe lack of healthcare personnel in wealthy nations, and the favorable recruiting policies with simple visa and work permit processes that draw our physicians away.^6^-^8^

Pakistan now has one of the worst rates of maternal and perinatal mortality in the world, as well as a threat from population explosion, extreme poverty, and a heavy burden of infectious and non-infectious illnesses. As a result, the social justice principle dictates that since Pakistan spends significant public resources to educate our doctors, it is reasonable to expect the graduates to “pay back” to their nation by fulfilling their roles for the greater goal of social good instead of the more limited goal of personal benefit.^9^ The purpose of this research was to determine how many medical students wish to leave Pakistan in quest of better prospects, the impact of this brain drain on the country’s healthcare system, and any potential preventive measures.

## METHODS

A cross-sectional study was conducted among (n=420) undergraduate medical students at two different medical colleges in Pakistan after approval from the institutional ethics review committee of Faisalabad Medical University No. (48.ERC/FMU/2022-23/267). Primary data was collected from medical students of all years from April to June 2022 by a structured questionnaire (combination of both open and closed questions) after the informed consent of participants. Non-probability sampling (Convenient Sampling) technique was used to collect the sample. The investigator himself collected the questions from the sample under study face to face. The data were analyzed by using the SPSS version 25. The frequency and percentage of data were calculated and presented in the results.

## RESULTS

Out of 420 medical students of both gender, 140 (33.3%) planned to leave Pakistan after graduation, while 280 (66.66%) were interested in pursuing careers in Pakistan.

**Fig.1:**
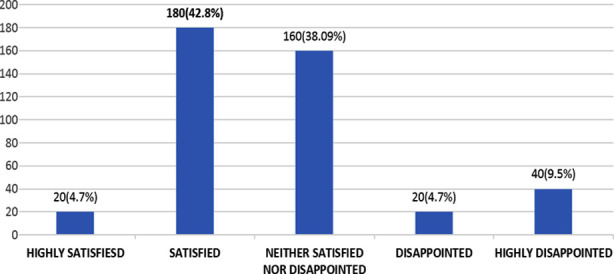
Satisfaction level with life in Pakistan.

When asked about the amenities offered to Pakistani doctors during their services, 20(4.7%) students report being highly satisfied, 180(42.8%) students report being satisfied, 160(38.09%) students report being neither satisfied nor disappointed, 20(4.7%) students report being disappointed, and 40(9.5%) students report being highly disappointed with life in Pakistan. The majority of students are satisfied with life in Pakistan (Fig.1).

However, when it came to medical students looking to immigrate, 220(52.38%) students think USA, 40(9.5%) students think UK, 40(9.5%) students think of Ireland, 120(28.57%) students think of Saudi Arabia is the best option for Pakistani doctors

**Fig.2:**
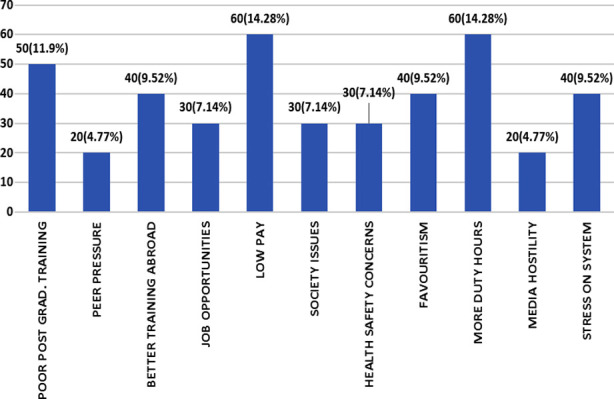
Causes of brain drain and its frequency.

**Fig.3:**
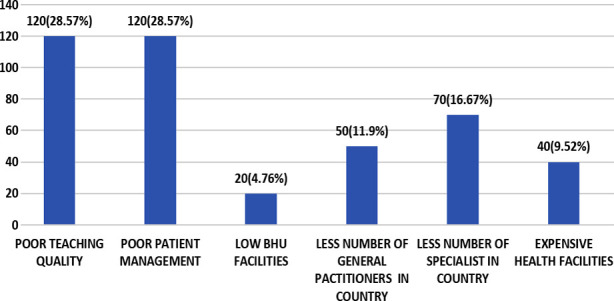
Effects of brain drain on Pakistan’s health care system.

The factors that influence doctors’ decisions to immigrate are highlighted in Fig-2 while the major effects of brain drain on Pakistan’s health system are highlighted in Fig-3.

Recommendations by the students to prevent brain drain, included, increase wages, reduce work cycles, improve service structure, improve the working environment, improve facilities materials, build more medical colleges, build more teaching hospitals, increase the number of places for PG training, change training styles and introduce modern methods to combat brain drain (Table-I).

**Table-I:** Recommendations made by medical students to prevent Brain Drain.

Recommendation	Frequency	Relative frequency %
Increase pay	70	16.67%
Decrease duty hours	30	7.14%
Better service structure	70	16.67%
Better working environment	30	7.14%
Improved facilities	30	7.14%
Build more medical colleges	20	4.76%
Build more teaching hospitals	30	7.14%
Increase seats of PG training	60	14.28%
Change in teaching style	20	4.76%
Adoption of modern methods	60	14.28%

## DISCUSSION

The brain drain in Pakistan has been the subject of several studies in the past. The primary objective of this research was to identify the reasons for migration and their influence on the healthcare systems, as well as potential remedies. Our sample had a 33% prevalence rate of migratory intents, which was lower than studies done at King Edward Medical College, Baqai Medical College, and Aga Khan University.^10^ The United States was the most popular migration destination among individuals who wanted to move abroad. This outcome is comparable to other studies done in Pakistan. This is due to the fact that the United States has some of the most beneficial laws for both temporary and permanent skilled migrants.^11^

There are better social and economic circumstances and there are more chances for work overseas. In developed nations, basic necessities like power, water supplies, health facilities, education, etc. are easily available. According to Act (8) of the Pakistani Constitution, everyone is allowed to migrate to any country in the world for life security. The vast majority of students agreed that physicians are increasingly vulnerable to insecurity and religious intolerance in Pakistan. In the last decade, doctors have been kidnapped for ransom, and as many as 32 people have been killed in sectarian killings.^12^

Pakistan produces 32,879 doctors per year, with 40% settling overseas. Pakistan now has 20,000 specialists. However, half of them are foreign nationals.^13^ Our study supports previous studies that 33% of students choose to study abroad due to a number of “push and pull factors” that seriously undermine Pakistan’s healthcare systems. Although there are a number of factors that influence doctors’ opinions on immigration, the majority of students believe that low pay and long hours are the biggest obstacles. While subpar patient care and trainee training are the main effects of brain drain on Pakistan’s health sector. The scenario is alarming for Pakistan’s health stakeholders.

### Limitations:

It include sampling methods, the study locations of two institutions and guidelines by universities’ seniors, personnel selection, and the socioeconomic status of each student included in the sample.

## CONCLUSION

One in three medical students plans to travel abroad after graduation due to a lack of resources and inadequate management in Pakistan. This has been adversely affecting the socio-medical situation and depriving Pakistan’s health system of essential medical services. Therefore, it is important for health policymakers in Pakistan to initiate campaigns to address the challenges faced by medical students and physicians in order to prevent brain drain.

### Abbreviations:

**IMG:** International Medical Graduate; **PG:** Post Graduation;

**BHU:** Basic Health unit; **HICs:** High-income countries;

### Authors’ Contribution:

**FN** conceived, designed and did statistical analysis & editing of manuscript, is responsible for integrity of research.

**FN, HS and HA** did data collection and manuscript writing.

**HS** did review and final approval of manuscript.
